# The Influence of Seasonality and Community-Based Health Worker Provided Counselling on Exclusive Breastfeeding - Findings from a Cross-Sectional Survey in India

**DOI:** 10.1371/journal.pone.0161186

**Published:** 2016-08-11

**Authors:** Aritra Das, Rahul Chatterjee, Morchan Karthick, Tanmay Mahapatra, Indrajit Chaudhuri

**Affiliations:** 1 CARE India Solutions for Sustainable Development, H No. 14, Patliputra Colony, Patna, Bihar, India; 2 Department of Epidemiology, Fielding School of Public Health, University of California Los Angeles, Los Angeles, California, United States of America; TNO, NETHERLANDS

## Abstract

**Background:**

Exclusive breastfeeding (EBF) during the first six months of life is considered a high impact but low-cost measure for reducing the morbidity and mortality among children. The current study investigated the association of seasonality and frontline worker(FLW) provided counselling with practice of EBF in Bihar, India.

**Methods:**

We used the ‘Lot Quality Assurance Sampling’ technique to conduct a multi-stage sampling survey in 8 districts of Bihar. Regarding EBF, mothers of 0–5 (completed) months old children were asked if they had given only breastmilk to their children during the previous day, while mothers of 6–8 (completed) months old children were inquired about the total duration of EBF. We tested for association between EBF during the previous day with season of interview and EBF for full 6 months with nursing season. We also assessed if receiving counselling on EBF and complementary feeding had any association with relevant EBF indicators.

**Results:**

Among the under-6 month old children, 76% received EBF during the previous day, whereas 92% of 6–8 (completed) months old children reportedly received EBF for the recommended duration. Proportion of 0–5 (completed) month old children receiving only breastmilk (during last 24 hours) decreased significantly with increasing age and with change of season from colder to warmer months. Odds of receiving only breastmilk during the previous day was significantly higher during the winter months (Adjusted odds ratio(AOR) = 1.50; 95% CI = 1.37, 1.63) compared to summer. Also, the children nursed primarily during the winter season had higher odds of receiving EBF for 6 months (AOR = 1.90, 95% CI = 1.43, 2.52) than those with non-winter nursing. Receiving FLW-counselling was positively associated with breastfeeding exclusively, even after adjusting for seasonality and other covariates (AOR = 1.82; 95% CI = 1.67, 1.98).

**Conclusions:**

Seasonality is a significant but non-modifiable risk factor for EBF. However, FLW-counselling was found to increase practice of EBF irrespective of season. Scale-up of FLW-counselling services, with emphasis on summer months and mothers of older infants, can potentially reduce the impact of seasonality on EBF.

## Introduction

Adequate nutrition, especially of the newborns, is a fundamental requirement for ensuring that the children realize their potential of developing into healthy adults. Globally, about 35% under-five mortalities are attributed to malnutrition.[1] A number of global and region-specific health interventions—such as universalizing early initiation of breastfeeding, breastfeeding exclusively during the first six months of life and age-appropriate frequency and quantity of complementary feeding from sixth month onwards—have focused on improving the nutritional status of children.[2, 3] In India, one of the key objectives of the ‘Integrated Child Development Services (ICDS)’ scheme has been to improve the nutritional status of children up to 6 years of age.[4] Unfortunately, in spite of such efforts, childhood nutritional indicators in India remained poor—with stunted, wasted and underweight children, respectively, accounting for about 48%, 20% and 43% of the total under-five year old population.[5] Amidst this grim premise, identification of the causes of childhood undernutrition and implementation of targeted measures to lessen its burden need to be prioritized.

Mother’s milk not only contains the essential nutrients required for optimal development but it is also digested easily by an infant.[6, 7] Further, the physical and the chemical compositions of mother’s milk evolve as the child matures—to meet the changing requirements of the growing infant.[8] World Health Organization (WHO) recommends that infants be given only breastmilk up to six month age and breastfeeding supplemented with semi-solid food should continue for another one and half years.[9, 10] Prior researches have asserted that promotion of exclusive breastfeeding (EBF) for the recommended duration could be a cost-effective policy tool for reduction in infant and under-five mortality, especially in the resource-limited settings.[11, 12] Moreover, benefits of breastfeeding are not only limited to children but also have important bearings on the mothers’ health. Some of the maternal benefits of early initiation and continuation of breastfeeding include reductions in postpartum bleeding, decreased risk of cancers e.g. breast and ovarian cancers, lesser likelihood of osteoporosis, and birth-spacing.[13]

Several factors that determine optimal EBF practices have been reported from India and other developing nations. These include cultural barriers, maternal education, age, parity, poverty, belonging to lower social strata/caste, burden of work on mothers, poor hygienic conditions, availability and uptake of antenatal and postnatal services, awareness about initiation and duration of breastfeeding.[14–18] In addition to these determinants, it has been hypothesized that the weather extremes associated with seasonal variations and the behavioral adaptations resulting from such weather changes can affect breastfeeding.[19–22] However, to the best of our knowledge, no Indian study has explored the impact of seasonal variation on EBF.

India has witnessed several measures aimed at reducing childhood morbidity and mortality. One of the foremost actions taken in this regard, under ICDS and National Rural Health Mission (NRHM) programs, was to equip a team of ground level health workers—known as the frontline workers (FLW)—for disbursing several interventions targeted at improving the maternal and child health. Besides providing tangible services like immunization and food support, FLWs (comprising of Anganwadi workers (AWW) and Accredited Social Health Activist (ASHA)) also provide various knowledge-based services e.g. counselling and awareness services.[23] Providing counselling services on infant feeding practices including EBF and complementary feeding, form an important part of FLWs’ job description.[24] As with seasonality, effectiveness of FLW-counselling on improving the uptake of EBF is yet to be evaluated.

India has made substantial progress towards achievement of its Millennium Development Goals (MDGs).[25] However, as we progress into the era of more ambitious Sustainable Development Goals (SDGs), achieving the targets pertaining to child survival and the prevention of malnutrition will require wider uptake of EBF.[26] The objectives of the current study were to investigate the impacts of seasonality and FLW-administered counselling on the EBF practices in an impoverished region of India. We expect our endeavor to inform the current and future programs on infant feeding in India.

## Methods

### Ethical approval

The current study was approved by the ‘Institutional Committee for Ethics and Review of Research’ of the Indian Institute of Health Management Research (www.iihmr.org), Jaipur, India. Informed consent (including signature or left thumb impression of the respondent) was obtained from each agreeing participant before the interview.

### Study population and setting

CARE India, a non-governmental organization, in association with the State Government of Bihar and under financial patronage of the Bill and Melinda Gates Foundation, initiated a multifaceted project named Integrated Family Health Initiative (IFHI) in 2011 with the principal objective of reducing the mortality and malnutrition among the infants and women of reproductive age in Bihar, a resource-poor state.[27] As part of the pilot phase of IFHI, multiple population based cross-sectional surveys were conducted to ascertain various health and developmental indicators in the state. In total, five rounds of such survey, using Lot Quality Assurance Sampling (LQAS) technique (a small sample survey design based on binomial distribution), [28–31] were conducted in eight randomly selected districts (from total 38) of Bihar. The sampling ‘lots’ in this survey were the blocks or sub-districts, which were the unit of programmatic intervention. In brief, using a multi-stage sampling strategy, 19 Anganwadi Center (AWC)–village level institutions providing basic health care services—areas were selected through probability proportional to size (PPS) sampling from each of the 137 blocks belonging to the eight randomly selected districts. From each selected AWC area, four eligible households were identified through systematic random sampling. The systematic random sampling of household involved the following steps:

1. The data collectors obtained an approximate number of households catered by an AWC from the *AWC Household Register* (or as per the information provided by an Anganwadi worker, where registers were not available)
2. Using a random number table, a random starting point—between 1 and the total number of households—was decided. This was considered the number of index household.
3. Counting from the location of AWC and following a ‘Right-hand rule’, the index household was reached. Starting from the index household, the data collectors visited every 5^th^ household and inquired if there was an ‘eligible mother’ in that household.
4. Eligible mothers were those that had a child belonging to any of the following age categories: 0–2, 3–5, 6–8, or 9–11 completed months.
5. The data collectors continued moving in a circular manner, following the ‘Right-hand rule’, till four interviews were conducted with eligible mothers (one from each age category).

Five iterations of above sampling strategy took place between October 2011 (LQAS Round I) and October 2014 (LQAS Round V). For each iteration of LQAS, altogether 2603 (137×19) AWCs were selected from the eight study districts and from the catchment areas of these AWCs, four households with children belonging to each of the four age sub-groups were identified. Consenting and eligible mothers responded to a detailed one-to-one interview conducted by trained study staff.

In the current analysis, we used information on infants belonging to three age groups—0–2, 3–5 and 6–8 completed months. The data obtained during Round-II to Round-V of LQAS survey were used. Information collected during Round-I of LQAS were excluded because of methodological shortcomings and changes in questionnaire pattern between the first and the subsequent rounds of survey.

### Sample size estimation

Our objective was to ensure adequate sample size for evaluation of various health indicators in each block (district sub-divisions). According to LQAS methodology, the suggested minimum sample size for each decision-making unit is 19 (resulting in <10% α and β errors).[32] Thus, based on the LQAS decision-making rule, 19 respondents per block for every age-group was selected.

### Outcome measures–breastfeeding indicators

During the interview, mothers of 0–2 and 3–5 completed months old infants were asked, inter alia, about the edible (or drinkable) items given to their child during the previous 24 hours (previous day’s morning to current day’s morning). Children of the mothers who reported giving only breastmilk were considered to be breastfed exclusively and vice versa.[33] Children who received other food or drinks including tea or other water-like substitutes (except for ORS or medicines) were regarded as non-exclusively breastfed. Thus, for less than 6 month old children, EBF status was determined by a surrogate measure based on 24-hour recall. Regarding feeding practice, if the mother was not available for some reason and some other lactating woman provided breastfeeding, it was still considered as breastfeeding. For the children aged 6–8 completed months, the mothers were inquired about the age at which weaning was done i.e. the age from which they started giving liquid/semi-solid food (other than breastmilk) to their child. Children who were reportedly weaned at or after sixth month were assumed to be exclusively breastfed.

### Predictors–seasonality and counselling services

In order to assess the effect of seasonal variation on breastfeeding among children aged 0–2 and 3–5 completed months, we first sought out the month during which the interview was conducted. Based on the prevailing weather pattern in Bihar, we classified the interviews conducted during November to February as those conducted in ‘winter’ season, April to August as ‘summer’ and rest of the months as ‘autumn/spring’.

For 6–8 month old children, we tried to determine the approximate calendar months during which an infant was supposed to be exclusively breastfed (as per WHO recommendation). In order to do this, we determined the months that comprised the six month period since their birth, counting from the month following the birth month of the child. For example, if a child was born in May, we conjectured that he/she was supposed to be exclusively breastfed from June to November. If three or more months of a child’s EBF period fell during the winter season (November to February), then his/her nursing season was categorized as ‘winter’, otherwise the nursing period was classified as ‘non-winter’.

As discussed previously, FLWs provide counselling on various maternal and child health issues including EBF and complementary feeding. As part of LQAS survey, mothers of children aged 0–5 months were asked if they received advice from FLWs regarding recommended EBF period. The mothers who reported receiving such advice following birth of the concerned child, were considered exposed to EBF counselling and vice versa. The mothers of older infants (6–8 completed months) were not inquired about EBF advice but they were asked about counselling on complementary feeding. The mothers who reported receiving advice on starting semi-solid food along with breast milk after their child reached a certain age (6 months) were classified as exposed to complementary feeding advice.

### Covariate measures

We included several demographic and socio-economic parameters in our analyses to control for potential confounding. These include gender of the child, caste, religion, economic status of the household (asset index), and parental education level. Caste-wise, the families were dichotomized into marginalized caste [scheduled castes (SC) / scheduled tribes (ST) / other backward castes (OBC)] and other/general caste. Religion categories were Hindu, Muslim, and other. According to the level of education, mothers and fathers were classified into three categories—no formal education, school education up to eighth standard, and school education above eighth standard. Economic status was assessed using an asset index (AI) based on possession of 25 different household items. For calculation of AI, a relative weight was assigned to each of these items (as per NFHS-3) [5] and an aggregated score was generated by adding the weighted score for each item possessed by a household. The cumulative asset scores were then log-transformed to create the AI. Based on the percentile distribution of AI, we then created AI tertiles and classified the families according to the AI tertile they belonged to—low, middle and high wealth. The calculation of asset index followed the methodology described by Kanungo et al.[34]

### Statistical analysis

Descriptive analyses were carried out to determine the distribution of socio-demographic and breast feeding related characteristics of the sampled population. For the purpose of descriptive and regression analyses, the datasets containing information on infants aged 0–2 months and 3–5 months were combined to create a concatenated dataset for 0–5 months old children; whereas the dataset on 6–8 month old children was treated as a separate entity.

We estimated the proportion of 0–5 month old infants, who were breastfed exclusively during past 24 hours in month-wise age groups, and further stratified the estimated proportions by season and FLW-counselling. We employed the Cochran-Armitage trend test to assess whether age-specific and/or seasonal trends existed in proportion of children receiving EBF. Among the children aged 6 to 8 months, we calculated the proportion of children who reportedly received EBF for the recommended duration—stratified by ‘winter’ and ‘non-winter’ nursing season. Further, we assessed the proportion of 0–5 month old infants who were breastfed exclusively during past 24 hours stratified by FLW-counselling. We also tested if there were any statistical differences in breastfeeding proportions between the children whose mothers received such advice and those who did not.

We implemented separate simple (crude) and multiple (adjusted) logistic regression models to identify determinants for the two dependent variables in our analyses—breastfeeding exclusively during past 24 hours (for 0–5 month olds) and EBF during the first 6 months of life (for 6–8 month olds). Further, only for the first dependent variable, in order to explore whether any age-specific differences existed in the association between ‘season of interview’ and breastfeeding exclusively during past 24 hours, six separate age-restricted models (one for each completed month in age—between 0^th^ and 5^th^ month) were employed. In the crude models for the first dependent variable, we separately tested associations with ‘season of interview’ and ‘FLW-provided EBF advice’, whereas ‘nursing season’ and ‘FLW-provided complementary feeding advice’ were the sole predictors in the unadjusted models for the second dependent variable. The adjusted models additionally included potential confounders—gender, religion, caste, economic status (AI tertile) and maternal education. All descriptive (*Proc surveymeans and Proc surveyfreq*) and associational (*Proc surveylogistic*) analyses were carried out using the survey data analysis procedures in SAS 9.4 and incorporated information about multi-stage sampling and relevant sampling weights.

## Results

The present analysis utilized data from 20793 mothers of 0–5 month old children and 10130 mothers of 6–8 month old children. Table 1 depicts the socio-demographic and breast feeding related characteristics of the study participants. We found that, in both 0–5 and 6–8 month age groups, about 86% participating families were Hindus. Only about 17% families lived in a ‘Pucca’ or brick-built house. Figures for parental educational status were quite poor—while about 26%-27% fathers studied beyond eighth standard, among mothers only 14%-15% had same level of education. About 76% mothers of 0–5 month olds reported that they breastfed their children exclusively during previous 24 hours. However, among the mothers of 6–8 month olds more than 92% claimed that they did not give any liquid/semi-solid food other than breastmilk during the first 6 months. Reported coverage of FLW-provided counselling on exclusive breastfeeding (mothers of 0–5 month olds) and counselling on initiation of complementary feeding (mothers of 6–8 month olds) were both about 31%.

**Table 1:** Socio-demographic and breast feeding related characteristics of children aged 0 to 8 completed months from 8 districts of Bihar. LQAS Survey: Rounds 2 to 5.

	Children aged 0–5 months (n = 20793)*		Children aged 6–8 months (n = 10130)*	
Characteristic	Frequency	Percent (%)	Frequency	Percent (%)
Age				
* 0 to <1 months*	3746	18.03	-	-
*1 to <2 months*	4027	19.22	-	-
*2 to <3 months*	2635	12.80	-	-
*3 to <4 months*	3981	19.36	-	-
*4 to <5 months*	3357	15.90	-	-
*5 to <6 months*	3047	14.69	-	-
*6 to <7 months*	-	-	3657	35.98
*7 to <8 months*	-	-	3119	30.66
*8 to <9 months*	-	-	3354	33.36
Religion				
*Hindu*	18247	86.67	8858	86.21
*Muslim*	2512	13.17	1247	13.52
*Christian*	28	0.13	18	0.19
*Jain*	1	0.01	2	0.02
*Others*	5	0.03	5	0.05
Caste				
*Scheduled caste*	5341	25.45	2657	26.07
*Scheduled tribe*	322	1.83	158	1.91
*Other backward castes*	12985	62.36	6271	61.69
*Others/General caste*	2145	10.37	1044	10.34
Type of house^1^				
*Kaccha*	8905	43.29	4297	43.01
*Semi-Pucca*	8376	39.98	4100	40.34
*Pucca*	3512	16.73	1733	16.65
Mother's education				
*No formal education*	13340	63.63	6586	64.44
*Studied up to 8th standard*	4262	20.99	2114	21.23
*Studied above 8th standard*	3182	15.38	1428	14.33
Father's education				
*No formal education*	8659	41.69	4350	43.01
*Studied up to 8th standard*	6234	31.47	2988	30.61
*Studied above 8th standard*	5487	26.84	2639	26.38
Family's asset index^2^				
*1st tertile (Low wealth)*	6785	32.57	3309	32.50
*2nd tertile (Middle wealth)*	6834	33.13	3357	33.58
*3rd tertile (High wealth)*	7174	34.29	3464	33.93
Was the child breastfed exclusively during past 24 hours?				
*Yes*	15896	76.33	-	-
*No*	4897	23.67	-	-
Was the child given anything other than breastmilk before the age of 6 months?				
*Yes*	-	-	712	7.32
*No*	-	-	9418	92.68
Did the mother receive advice on duration of exclusive breastfeeding from FLW?^3^				
*Yes*	6708	30.81	-	-
*No*	9610	46.84	-	-
*Do not remember*	4475	22.35	-	-
Did the mother receive advice on age of initiation of complementary feeding from FLW?^3^				
*Yes*	-	-	3259	30.51
*No*	-	-	2304	22.38
*Do not remember*	-	-	4567	47.11

*Observations with missing values excluded as applicable.

^1^Type of house: 'Kaccha'—house made of mud, grass, bamboo, thatch and other low quality materials.; 'Pucca'—structure made of brick; 'Semi-Pucca'—any combination of the components of 'Kaccha' and 'Pucca' houses.

^2^Based on possession of 25 different household items.

^3^FLW consists of Anganwadi workers (AWW), Accredited Social Health Activist (ASHA) and Auxiliary Nurse Midwives (ANM).

**Fig 1:**
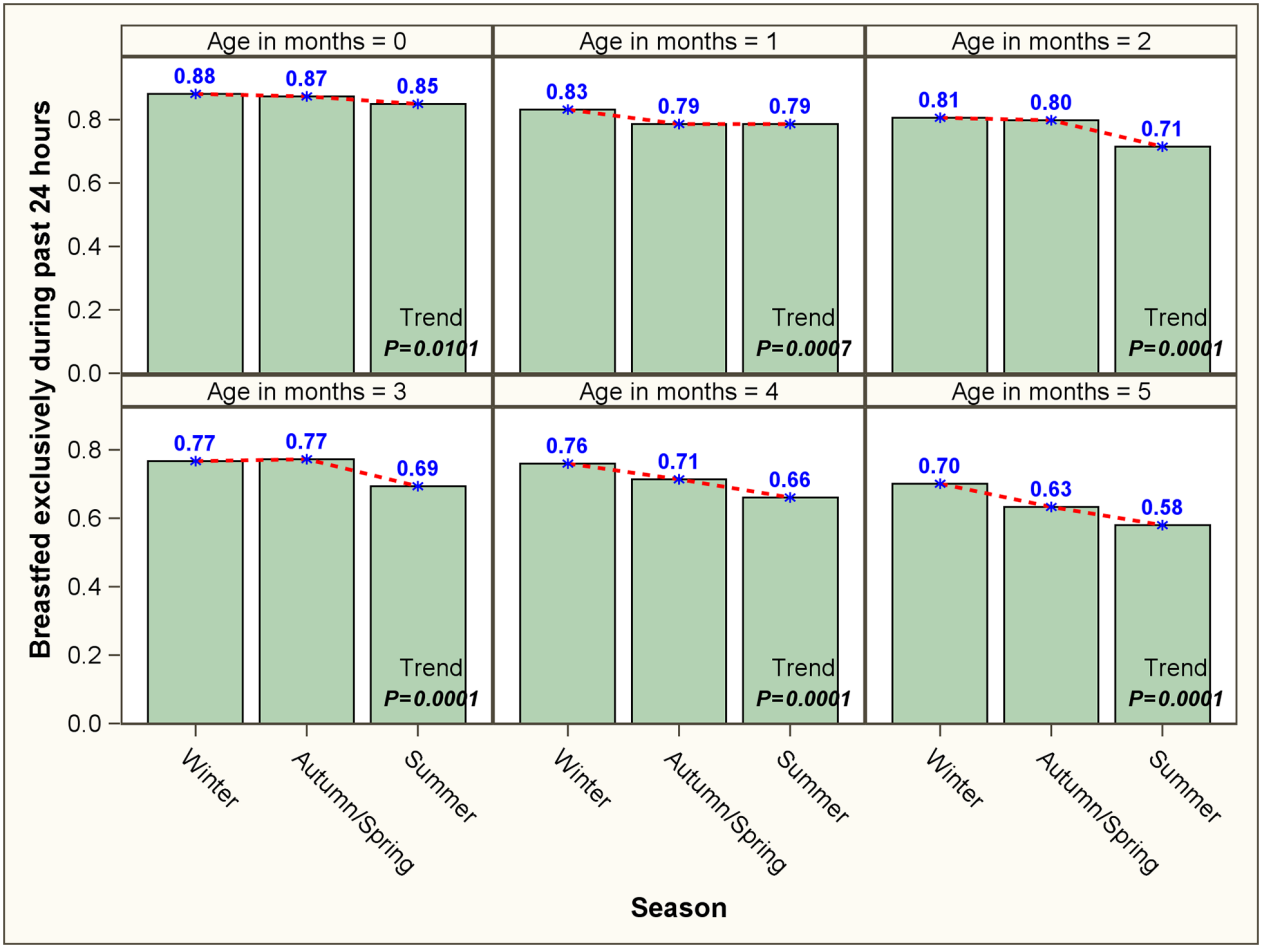
Season-wise trend in the proportions of 0–5 completed months old children who received only breastmilk during the previous 24 hours, stratified by age.

**Fig 2:**
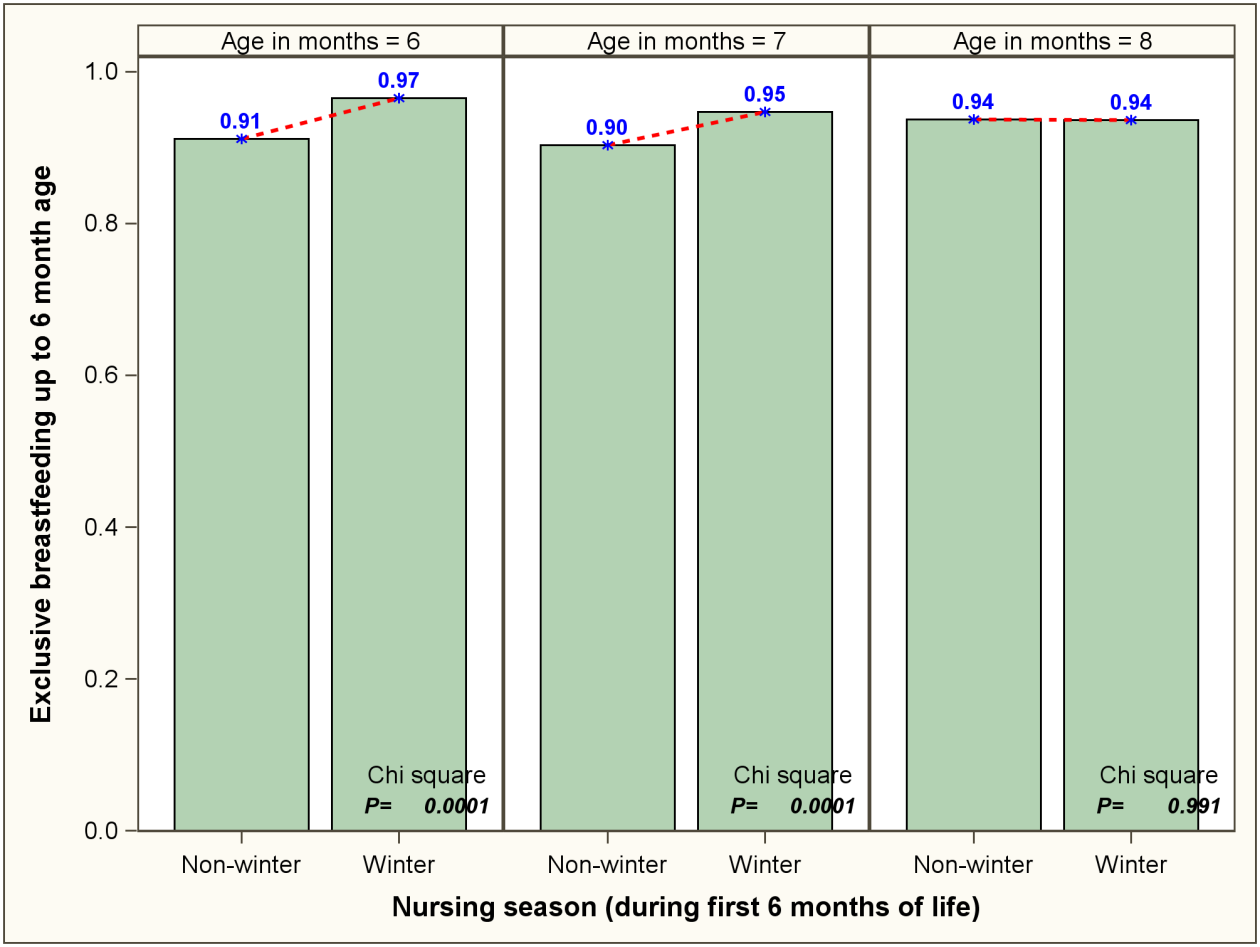
Proportions of 6–8 completed months old children who were exclusively breastfed for 6 months, stratified by ‘season of nursing’.

**Fig 3:**
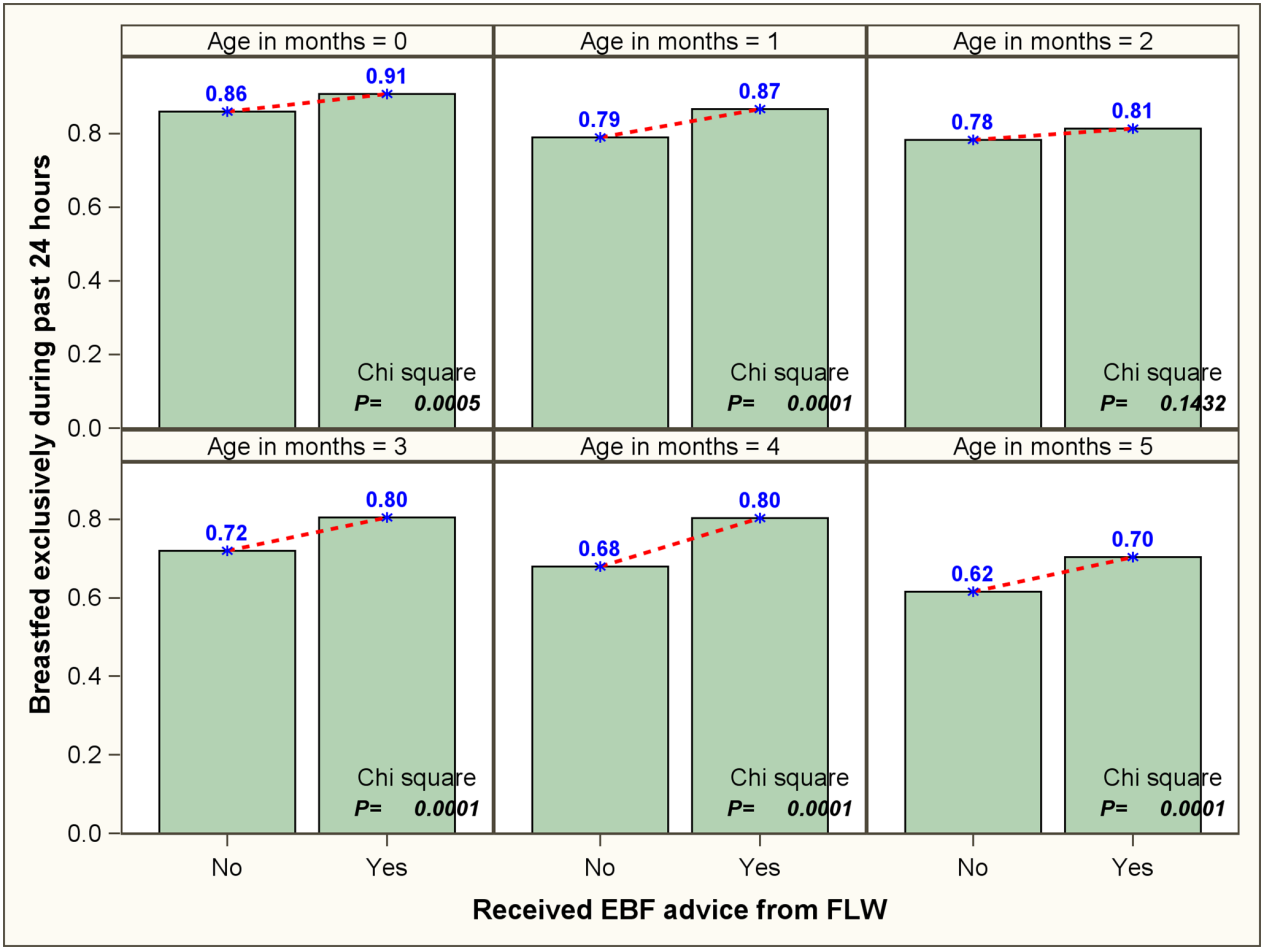
Proportions of 0–5 completed months old children who received only breastmilk during the previous 24 hours, stratified by age and exposure to counselling on exclusive breastfeeding.

Proportion of children who were breastfed exclusively during past 24 hours was about 87% for neonates (<1 month old). However, a significant decreasing trend (P<0.0001) was seen in this proportion during the subsequent months of life, with 64% children older than 5 months receiving only breastmilk during the previous day (Table 2). Fig 1 depicts the proportion of 0–5 month old children, stratified by age, who received only breastmilk during past 24 hours. A statistically significant decreasing seasonal trend was noticed in each age group with proportion of children receiving only breastmilk being highest during the winter months, followed by autumn/spring and summer. Among the children in their sixth and seventh month, it was found that the reported EBF during the first six months was higher for children receiving ‘winter nursing’ as compared to ‘non-winter nursing’; however, no difference was noted for the >8 month age category (Fig 2). We also found that the children (0–5 month old) whose mothers received counselling on EBF had significantly higher probability of receiving only breastmilk during last 24 hours, irrespective of age group (Fig 3).

**Table 2:** Proportion of children aged between 0–5 completed months who were breastfed exclusively during past 24 hours, according to age group. LQAS Survey: Rounds 2 to 5. (n = 20793).

Age in month	Estimated proportion (%)	95% CI of predicted %	Cochran-Armitage Trend Test	
			Statistic	P-value
0 to <1 months	86.52	85.45, 87.58	23.48	<0.0001
1 to <2 months	81.61	80.48, 82.74		
2 to <3 months	77.15	75.63, 78.66		
3 to <4 months	74.34	73.05, 75.63		
4 to <5 months	71.49	70.02, 72.95		
5 to <6 months	64.06	62.46, 65.66		

Table 3 provides the crude and adjusted associations of EBF with seasonal variations among children aged 0–5 and 6–8 completed months. Overall, among the 0–5 month olds, during both autumn/spring (adjusted odds ratio (AOR) = 1.30; 95% confidence interval (CI) = 1.16, 1.46) and winter seasons (AOR = 1.50; 95% CI = 1.37, 1.63) children had significantly higher odds of receiving only breastmilk compared to summer. When we stratified the 0–5 month olds into narrower monthly age groups, we found that the adjusted associations of season with breastfeeding exclusively were significant only among the older age categories. In the 6–8 month age group, we tested the association of nursing during the winter months with EBF during the first six months. It was found that the children for whom ≥3 months of EBF period fell during the winter season had significantly higher odds of receiving EBF (AOR = 1.90, 95% CI = 1.43, 2.52).

**Table 3:** Crude and adjusted associations of exclusive breastfeeding with seasonal variations among children aged 0–5 and 6–8 completed months. LQAS Survey: Rounds 2 to 5.

Outcome indicator	Age groups	Predictor	Predictor categories	Unadjusted		Adjusted^1^	
*Children aged 0–5 completed months (n = 20793)*				OR	95% CI	AOR	95% CI
Exclusive breastfeeding of the child during past 24 hours [Reference = 'No']	0 to <1 months	Season during which interview was conducted [Reference = 'Summer']	Autumn/Spring	1.11	0.72, 1.70	1.09	0.71, 1.67
			Winter	1.48*	1.07, 2.05	1.37	0.98, 1.91
	1 to <2 months		Autumn/Spring	0.91	0.65, 1.28	0.85	0.61, 1.20
			Winter	1.15	0.88, 1.52	1.01	0.76, 1.33
	2 to <3 months		Autumn/Spring	1.5	0.95, 2.36	1.48	0.93, 2.35
			Winter	1.36*	1.01, 1.82	1.31	0.97, 1.77
	3 to <4 months		Autumn/Spring	1.51*	1.19, 1.91	1.47*	1.15, 1.87
			Winter	1.56*	1.32, 1.85	1.47*	1.23, 1.75
	4 to <5 months		Autumn/Spring	1.42*	1.11, 1.80	1.28*	1.00, 1.64
			Winter	1.67*	1.40, 2.00	1.53*	1.28, 1.84
	5 to <6 months		Autumn/Spring	1.29*	1.02, 1.64	1.24	0.97, 1.57
			Winter	1.81*	1.52, 2.15	1.75*	1.47, 2.09
	All age groups		Autumn/Spring	1.37*	1.22, 1.54	1.30*	1.16, 1.46
			Winter	1.64*	1.50, 1.78	1.50*	1.37, 1.63
*Children aged 6–8 completed months (n = 10130)*							
Exclusive breastfeeding up to 6 month age of the child [Reference = 'No']	All age groups	Nursing season (If ≥3 months of a child’s recommended EBF period fell during winter season) [Reference = 'Non-winter']	Winter	1.89*	1.42, 2.50	1.90*	1.43, 2.52

^1^Adjusted for gender, religion, caste, economic status (asset index), mother's education. The model for children aged 6–8 months was also adjusted for variation in age. Further, all the models for 0–5 months old children were adjusted for receiving breastfeeding advice from FLW and the model for children aged 6–8 months adjusted for receiving complementary feeding advice from FLW.

*Statistically significant (p<0.05).

Table 4 presents the crude and adjusted associations of receiving only breastmilk during past 24 hours with FLW-counselling among 0–5 month old children. The relationship between EBF for first six months of life and counselling on initiation of complementary feeding (for 6–8 month olds) is also depicted on the same table. It was found that, overall, receiving FLW-counselling on EBF was a significant positive predictor of breastfeeding exclusively during past 24 hours (AOR = 1.82; 95% CI = 1.67, 1.98). In the age subgroup analyses, for each completed month in age, the above association remained significant except for children in the second month of life. On the other hand, receiving advice on timely initiation of complementary feeding was found to be a significant negative determinant of EBF in the unadjusted analysis (OR = 0.79; 95% CI = 0.62, 1.00). The negative association persisted in the adjusted analysis but the association was no longer statistically significant (AOR = 0.85; 95% CI = 0.66, 1.08).

**Table 4:** Crude and adjusted associations of exclusive breastfeeding with FLW-provided counselling on exclusive breastfeeding (for mothers of 0–5 month olds) and on timely initiation of complementary feeding (for mothers of 6–8 month olds). LQAS Survey: Rounds 2 to 5.

Outcome indicator	Age groups	Predictor	Unadjusted		Adjusted^1^	
*Children aged 0–5 completed months (n = 20793)*			OR	95% CI	AOR	95% CI
Exclusive breastfeeding of the child during past 24 hours [Reference = 'No']	0 to <1 months	Receiving advice from FLW on exclusive breastfeeding [Reference = 'No']	1.54*	1.15, 2.07	1.48*	1.10, 2.00
	1 to <2 months		1.74*	1.37, 2.19	1.84*	1.45, 2.34
	2 to <3 months		1.19	0.90, 1.58	1.18	0.89, 1.57
	3 to <4 months		1.57*	1.32, 1.87	1.54*	1.29, 1.84
	4 to <5 months		1.83*	1.52, 2.20	1.87*	1.54, 2.26
	5 to <6 months		1.45*	1.22, 1.74	1.40*	1.17, 1.68
	All age groups		1.84*	1.70, 2.00	1.82*	1.67, 1.98
*Children aged 6–8 completed months (n = 10130)*						
Exclusive breastfeeding up to 6 month age of the child [Reference = 'No']	All age groups	Receiving advice from FLW on timely initiation of complementary feeding [Reference = 'No']	0.79*	0.62, 1.00	0.85	0.66, 1.08

^1^Adjusted for gender, religion, caste, economic status (asset index), and mother's education. The model for children aged 6–8 months was also adjusted for variation in age. Further, all the models for 0–5 month old children were adjusted against the season during which the interview took place and the model for children aged 6–8 months adjusted for the season during which most of the nursing (in first 6 months) took place.

*Statistically significant (p<0.05).

## Discussion

Exclusive breastfeeding is widely recognized as an inexpensive, contamination-free, immunity boosting and emotionally satisfying way of providing adequate nutrition to infants during the first half year of life, especially in resource-poor settings. Our results show that more than 81% children in their second month of life were breastfed exclusively during the past 24 hours. This was much higher than that reported from a nationally representative survey conducted in 2005–06.[5] The increased proportion observed in this study could be attributed to the breastfeeding awareness campaigns that might have improved acceptability of EBF over the years. Moreover, as mentioned before, the current study was conducted in one of the most impoverished regions of India. It could be the case that even if some of the mothers wanted to substitute or supplement breastmilk, which is free, economic constraints might have prevented them from doing so.[20, 35] The reported prevalence of EBF for WHO recommended duration (i.e. 6 months) was also found to be higher in the current study compared to previous findings from India.[36–39] However, we must admit that the information on EBF for full six month duration has lesser validity than the data on breastfeeding during past 24 hours. The data on EBF for 6 months (based on long recall period) is prone to greater amount of recall bias and depends heavily on the mothers’ ability to determine the age of the child accurately—an unlikely scenario given the poor educational status of the participating mothers. Still, bearing in mind the prevailing socio-economic situation, it seems likely that the proportion of mothers practicing EBF is greater in Bihar compared to the national average. We also observed that the practice of breastfeeding exclusively (24 hour recall) gradually declined with increasing age of the child. This decreasing trend was similar to that reported in National Family Health Survey (NFHS-3) and also in a study from neighboring Bangladesh.[5, 33]

Our analysis revealed existence of significant association between seasonality and exclusivity of breastfeeding among 0–5 month old children—overall and within each age strata. It was found that babies were most likely to be exclusively breastfed during the winter season, followed by autumn/spring and summer. Moreover, children who got winter nursing were more likely to receive EBF for recommended duration compared to those who were nursed in non-winter months. Although there has been no Indian study in this regard, evidence from Africa, Latin America and Europe indicate that seasonal variations can indeed influence breastfeeding patterns.[19–22] As a potential explanation, we hypothesize that during the warmer months mothers perceive that their children remain thirsty despite breastfeeding.[40] Moreover, as noted in a study from Bangladesh, summer is the pre-harvesting season in the tropical South-Asia.[41] Therefore, in the poorer strata of the society harsher summer months are often associated with lack of adequate maternal nutrition (due to limited availability of food) and weight loss. We postulate that diminution in the nutrition status of self may lead mothers to perceive that their children are not getting adequate nutrition and, in turn, prompt them to supplement breastfeeding with additional liquid/semi-solid food.[41] Our results also indicate that the impact of seasonality on breast feeding is more pronounced in the latter half of the first six months of a child’s life. This could be due to the fact that the mother’s concerns about her child’s lack of adequate hydration or nutrition during the hotter seasons get more pronounced with the physical growth and increasing nutritional demands of the infant.

A marked increase in practice of EBF has been observed in nations that have implemented some supportive measures for lactation.[3] Further, evidence from developing countries around the world suggest that counselling by community health workers can be an effective strategy to improve infant feeding practices including EBF.[33, 42–45] In the current study, too, EBF among 0–5 month old children was positively associated with receiving advice from FLW on the same. Surprisingly, practice of EBF for full six months was found to be lower among mothers who received counselling on initiation of complementary feeding, compared to those who did not. There could be two probable explanations for such a finding. First, being advised on rationale of complementary feeding might make some mothers apprehensive about the nutritional adequacy of EBF. The poor educational status of participating mothers might also have contributed to this misperception. Second, this finding could be an artifact of reverse causation. Unlike EBF counselling, counselling on complementary feeding is likely to be provided when the child is already few months old. The ‘defaulter’ families i.e. the families with poorer child health indicators and practices (e.g. infant feeding practices, immunization) might get identified by local Anganwadi Center during initial months of a child’s life. Thus, a spurious negative association between complementary feeding advice and EBF could have arisen from the fact that the FLWs were more focused on visiting the families that did not follow the recommended practices during the initial phase of a child’s life.

Seasonality is a natural phenomenon and, for obvious reasons, not modifiable by simpler means. Consequently, lessening the impact of seasonal variation on EBF may prove difficult. Nevertheless, we observed that even after controlling for seasonal variation FLW-counselling showed significant positive association with practice of EBF. Therefore, we would like to endorse widespread implementation of EBF promotion through counselling by FLW and other community level health workers—a strategy with proven efficacy in reducing infant morbidity and mortality.[33, 43–45] Moreover, as NRHM and ICDS programs already include provision for the same, this strategy might prove cost-effective from a programmatic perspective as well. We opine that the concerned program(s) can be strengthened with following modifications—a) intensification of FLW-provided EBF counselling activities during the summer months, and b) emphasis on reaching the mothers of older infants (3–5 months) for maintenance of EBF. Moreover, among our study participants only about 31% mothers reported ever receiving advice from FLW. Therefore, coverage of FLW-counselling need to be increased.

There were some important limitations in the current study. First, owing to the cross-sectional design, we were often unsure about the temporal relation between the study parameters. This limited our ability to draw causal inferences from the observed associations between the dependent and predictor variables. Second, the information on breastfeeding practices were based on mother’s report and not actual observation. Thus possibility of social desirability bias—the mothers who were aware about EBF might have reported that they practiced the same even if they did not—cannot be ignored. Also, information on whether the baby was breastfed by the biological mother or someone else was not collected. The reported nature of the data also made our analyses susceptible to recall bias—especially for the data on EBF during the first six months. The proportion of children receiving recommended duration of EBF were based on interview of mothers of 6–8 month old children. It is evident that there was some degree of measurement error (over-reporting of EBF) regarding this parameter, as proportion of mothers who reported practicing EBF for full six months was higher than the proportion of mothers who breastfed exclusively during the previous 24 hours. Also, lack of information on the quality and intensity of the EBF counselling could be a potential source of information bias. Still, recall of receiving such counselling might actually have worked as a filter for only those interactions with adequate intensity because of which those were perceived as relevant inputs. Finally, as mentioned before, the current study was conducted in an economically backward region of India. The poor people are likely to be more vulnerable to the natural events including seasonal variation. Therefore, our findings may not be generalizable to pan-India level and also among families belonging to higher socioeconomic strata.

Despite the limitations, our study benefitted from the fact that we used a validated survey sampling design to collect information on a large study population. The large sample size afforded us to control for multiple covariates simultaneously in the regression analyses and to perform age subgroup analyses. Moreover, as the LQAS surveys were conducted across multiple rounds during different times of the year, we could analyze the seasonal trends without being concerned about the sample size. Additionally, a uniform protocol and rigorous training methodology was implemented across the survey regions and rounds, which helped us reduce the between-interviewer variations and improve the quality of collected data.

Promotion of EBF for recommended duration has been recognized as a key intervention to reduce the childhood malnutrition and mortality in India.[46] Findings of the current study suggest that practice of EBF is associated with seasonality, a non-modifiable risk factor. On the other hand, FLW-provided counselling was found to have a positive association with EBF, irrespective of the seasonal variations. Therefore, we recommend addressing the potential inclination towards variation in practice of EBF owing to seasonality during counselling for EBF and increasing the coverage of such counselling to improve the uptake of EBF. A carefully designed community-based cluster-randomized trial can overcome the methodologic limitations of the present study and provide more robust evidence on the relation between seasonality, FLW counselling and EBF. Such trials can further help in identifying the potential pathways by which weather can affect infant feeding practices. Moreover, qualitative exploration of mothers’ perceptions and practices about breastfeeding during the different seasons may also prove helpful in this regard.
